# Draft Genome Sequence of *Staphylococcus aureus* 4185, a Strain That Produces Aureocyclicin 4185

**DOI:** 10.1128/genomeA.01249-17

**Published:** 2017-11-02

**Authors:** Márcia Silva Francisco, Felipe Miceli Farias, Ilana Nascimento Sousa Santos, Selda Loase Salustiano Marques-Bastos, Rodolpho Mattos Albano, Maria do Carmo Freire Bastos

**Affiliations:** a Departamento de Microbiologia Geral, Instituto de Microbiologia Paulo de Góes, Universidade Federal do Rio de Janeiro, Rio de Janeiro, Brasil; b Departamento de Bioquímica, Instituto de Biologia Roberto Alcântara Gomes, Universidade do Estado do Rio de Janeiro, Rio de Janeiro, Brasil

## Abstract

The draft genome sequence of the aureocyclicin 4185-producing strain *Staphylococcus aureus* 4185 is presented. The assembly contains 2,789,721 bp and a G+C content of 32.8%. Genome analysis allowed us to determine the complete sequence of the bacteriocinogenic plasmid pRJ101 and to find another bacteriocin gene cluster encoded on the bacterial chromosome.

## GENOME ANNOUNCEMENT

*Staphylococcus aureus* 4185, a strain isolated from bovine mastitis in Brazil, exhibits potential biotechnological applications due to its antimicrobial activity against important pathogens (1), which is provided by the production of two antimicrobial peptides (2). One of them, named aureocyclicin 4185, is encoded on plasmid pRJ101 and seems to be a cyclic peptide, the first one described in staphylococci (3). However, this peptide is found at very low levels in the culture supernatant of the pRJ101 host strain (3). It is known that bacteriocin production is usually well controlled by the cell and that regulatory genes are found as either part of the bacteriocin locus or not (4, 5). Most of the pRJ101 sequence was previously determined, except for ~100 bp, with a strong secondary structure. As pRJ101 does not carry genes involved in regulation of bacteriocin production (3), these genes might be encoded on the bacterial chromosome. Therefore, sequencing of the bacterial genome was expected (i) to provide the complete nucleotide sequence of pRJ101 and to contribute to finding (ii) the regulatory genes involved in aureocyclicin 4185 production and/or (iii) a second bacteriocin gene cluster.

The sequencing library was constructed using the Nextera XT DNA library preparation kit (Illumina), following the manufacturer’s recommendations. Whole-genome shotgun sequencing was performed on the Illumina MiSeq system with the 500-cycle MiSeq reagent v2. *De novo* assembly of 2,497,766 paired-end reads was done using the A5-miseq pipeline (6), yielding >300-fold average genome coverage and resulting in a draft genome comprising 29 scaffolds ranging from 537 to 515,431 bp. The final assembled genome was shown to have 2,789,721 bp, featuring a G+C content of 32.8%.

Genome annotation was performed by the Rapid Annotations using Subsystems Technology (RAST) server (7), which found 2,625 coding sequences and 80 RNA sequences. Most genes were related to amino acid, protein, carbohydrate, and RNA metabolisms and biosynthesis of cofactors, vitamins, and prosthetic groups. Genes involved in resistance to teicoplanin, fluoroquinolones, and heavy-metal ions and production of beta-lactamases and multidrug resistance efflux pumps were also found. Genes encoding microbial surface components recognizing adhesive matrix molecules and biofilm formation were identified as well. The PHAST server (8) identified seven regions containing prophage sequences, with three complete prophages, which may be involved in the regulation of aureocyclicin 4185 production (5).

The search for plasmid replicons was performed by analyzing all scaffolds for genes encoding plasmid Rep proteins. Plasmid pRJ101 was identified in scaffold 21, and together with our previous data, it was possible to determine its complete sequence of 11,653 bp, including the aureocyclicin 4185 gene cluster. Additionally, by using the BACTIBASE database (9) and visual inspection, a putative bacteriocin gene cluster was found on the chromosome of *S. aureus* 4185 (in scaffold 4), which seems to encode a new class II staphylococcin, to be further characterized.

### Accession number(s).

This whole-genome shotgun project has been deposited at DDBJ/ENA/GenBank under the accession no. NWUN00000000. The version described in this paper is the first version, NWUN01000000.
